# How Swift Is Cry-Mediated Magnetoreception? Conditioning in an American Cockroach Shows Sub-second Response

**DOI:** 10.3389/fnbeh.2018.00107

**Published:** 2018-05-28

**Authors:** Pavel Slaby, Premysl Bartos, Jakub Karas, Radek Netusil, Kateřina Tomanova, Martin Vacha

**Affiliations:** Faculty of Science, Institute of Experimental Biology, Masaryk University, Brno, Czechia

**Keywords:** magnetoreception, Cryptochrome, conditioning, transduction time, insect, inter-stimulus interval

## Abstract

Diverse animal species perceive Earth’s magnetism and use their magnetic sense to orientate and navigate. Even non-migrating insects such as fruit flies and cockroaches have been shown to exploit the flavoprotein Cryptochrome (Cry) as a likely magnetic direction sensor; however, the transduction mechanism remains unknown. In order to work as a system to steer insect flight or control locomotion, the magnetic sense must transmit the signal from the receptor cells to the brain at a similar speed to other sensory systems, presumably within hundreds of milliseconds or less. So far, no electrophysiological or behavioral study has tackled the problem of the transduction delay in case of Cry-mediated magnetoreception specifically. Here, using a novel aversive conditioning assay on an American cockroach, we show that magnetic transduction is executed within a sub-second time span. A series of inter-stimulus intervals between conditioned stimuli (magnetic North rotation) and unconditioned aversive stimuli (hot air flow) provides original evidence that Cry-mediated magnetic transduction is sufficiently rapid to mediate insect orientation.

## Introduction

Animals with a vast diversity of lifestyles and types of locomotion perceive the Earth’s magnetic field (reviewed e.g., in Wiltschko and Wiltschko, 2006). Migrating species use the direction of the geomagnetic field (GMF) to find and keep the bearings of their routes in the same way a person holding a magnetic compass does. Some of them, like birds, turtles, eels and also the invertebrate lobster can employ other GMF parameters and localize their positions during migration like a man using the GPS system (Lohmann et al., 2007). However, even non-migrating invertebrates like the fruit fly, cockroach, honeybee or marine *Decapoda* (reviewed in Vacha, 2017) are equipped with a magnetic sense. Detecting the magnetic vector turns out to be indispensable for orientating animals traveling no more than a few kilometres, as a maximum (Wyeth, 2010).

Recent molecular studies have shown that the fruit fly’s and American cockroach’s magnetic sensitivity (Gegear et al., 2008; Bazalova et al., 2016), respectively is mediated by flavoprotein Cryptochrome (Cry). The evidence of its role as a proximate magnetic receptor has definitely not been achieved *in vivo* yet, but both experimental chemical studies *in vitro* (Maeda et al., 2008) and theoretical physical analyses and simulations (Lau et al., 2012; Hiscock et al., 2016) make Cry the most likely candidate for a chemical magnetoreceptor. It is thought to create—in partnership with its cofactor FAD—a radical pair (RP) which confers sensitivity to the GMF on the whole molecule (reviewed e.g., in Hore and Mouritsen, 2016). According to the RP reception model, upon FAD being activated by blue light, it passes through the cycle of several redox states. Some of these possibly activate Cry into the signaling state. The GMF direction may control the transitions between the cycle steps, hence controlling the Cry signaling as well. The precise steps from activation of Cry to subsequent pathways which send biologically relevant signals to the nervous system mediating behavioral responses are not yet known. Cry’s conformational change may be expected, along with messenger’s downstream involvement (Hore and Mouritsen, 2016) impacting ion channel gating and finally resulting in change of membrane potential as was shown on a potassium channel by Fogle et al. (2015).

Chemical magnetoreception should be swift enough to be able to control movement and orientation in animals, which are both speedy and occupying relatively small niches as in the case of insects. Hence, the delay between the magnetic stimulus and neural response must be comparable to other classical senses like vision, smell or touch. All of the still speculative models on how the Cry’s biochemical activity change may lead to changes in the membrane potential of sensory neurons should take this time aspect into consideration. Although it is an important part of the RP hypothesis, the rapid conversion of GMF energy into change of receptor membrane potential has never been verified.

The electrophysiology discipline is at the forefront in revealing the transduction time and processing time of any sensory information most competently. However, available electrophysiological records on neural response speed to magnetic stimuli differ. Cases of stimulus-response delay taking up to tens of minutes are surprisingly often in literature (discussed in Wang et al., 2004; Pavlova et al., 2011). Latencies such as this would be uneasy to reconcile with rapid movement control.

In our previous behavioral work, we also noticed the delay of onset and the persistence of spontaneous movement reaction to experimental 60° shifts of the GMF axis, which took up to tens of minutes in the American cockroach *Periplaneta americana* (Vácha, 2006). Similar reports of magnetic orientation delays in invertebrates exist (e.g., Lohmann et al., 1995 or Bae et al., 2016). Recently, we have found that *Periplaneta* magnetoreception is dependent on mammalian-like Cry 2 (Bazalova et al., 2016). Consequently, we wondered how swift behavioral response to magnetic stimulus mediated by Cry could be.

In this work we set out from the observations that delayed spontaneous behavioral reaction following the magnetic signal may be almost arbitrarily long and does not reflect the transduction speed in naïve animals. Using aversive conditioning, we developed a reaction of temporary locomotor activity drop to magnetic stimulus (magnetically induced freezing, MIF) in the American cockroach. By means of a series of various inter-stimulus intervals (ISIs) we show that neural processing of magnetic cue takes place within a time frame of less than a second. This work provides original behavioral evidence that Cry-mediated magnetoreception is swift enough to control animal locomotion.

## Materials and Methods

We adopted the basic design of our previous paradigm testing unconditioned movement reaction of an American cockroach on periodic natural GMF rotations (Vácha, 2006). However, since we focused on the time necessary for transducing and neural processing of magnetic stimulus, we switched to a conditioning paradigm now. We have utilized the fact that the time gap between subsequent sensory cues (ISI) defines if and how a conditioned link between them is made. Especially if unpleasant, potentially harmful stimuli are used for conditioning (aversive conditioning) the time spent by magnetic transduction and transmission must roughly be equal to or lesser than the shortest functional ISI (see “Discussion” section). In our recent work, the time between GMF 60° rotations (conditioned stimulus—CS) and the onset of the hot air stream (unconditioned stimulus—US) were changed so that the shortest functional ISI was defined.

### Experimental Room

The experiments took place between 2011 and 2018 in an empty brick building behind the university campus where the risk of any disturbance was minimized. A 12 h light and 12 h dark regimen with a temperature between 21–25°C was maintained in the experimental room. Noise and vibrations were monitored by seismometer (Seismic Accelerometer, Wilcoxon Research, Model 731A, Wilcoxon, Power unit Amplifier Model P-31, Audacity 1.3 software) and the data were discarded prior to evaluation if any vibrations and noises occurred during the experiment (12 cases). The computer controlling frame capture and stimuli applications was located in a neighboring room so that no personnel dwelled in the building in order to avoid possible disturbances during the experiments. The experiments were designed as blind and the staff involved in conducting the experiments had no information about what type of experiment was being prepared and evaluated.

### Magnetic Conditions

A single double-wrapped Merritt coil (2 × 2 × 2 m) made of wood made it possible to control the direction of the horizontal GMF component. Double wrapping enables the coil to be fed with full current without MF generation. This way, controlling non-specific effects possibly accompanying electrically generated fields can be performed. The total vector of local GMF intensity was 46.8 uT (homogeneity ± 0.2 µT) and inclination 68° on the testing table (measured using a FGM3D probe, Sensys). The Merritt coil’s magnetic axis made a 120° angle with horizontal GMF and feeding the coil with current 1.05 A rotated the North position by 60°, where the change in inclination and intensity of the final rotated field vector was less than 1%. A custom made programmable power source controlled the currents feeding the coil during GMF rotations. Both onset and decline of the current from 0 A up to 1.05 A and back were linear and took 5 s. Periods of natural steady field and rotating field (RF) alternated. No current entered the coil during the steady period and GMF rotated back and forth by 60° alternating every 5 min during the RF period.

Radiofrequency background was not shielded. Spectrum analyzer (FSC3, 9 kHz–3 GHz, Rohde and Schwartz) and calibrated 6511 Lindgren passive antenna for magnetic component and active antenna Schwarzbeck EFS 9218 for electric component were used to measure the local magnetic radiofrequency noise. A representative sample and details of settings are in Supplementary Figure S1.

### Experimental Setup and Schedule

The animals (American cockroach, *Periplaneta americana*) were bred in plastic containers under 12 h light, 12 h dark conditions with an ambient temperature of 23°C in the rearing room. Individuals regardless of sex were chosen for each trial and kept individually in Petri dishes (diameter 15 cm). To allow the hot air flow in, grids made of wire were used as dish lids; a ring of paper tape around the dishes eliminated visual contact among animals. The experiment was split into training and testing on the next day.

Both training and testing were performed in the same arena, made of white plastic (diameter of 60 cm and height of 40 cm). The arena was placed on the wooden table in the center of the Merritt coil (Supplementary Figure S2). The animals were placed into the arena at least 12 h before the training. The dishes with the animals were arranged along the inner perimeter of the arena, while a plastic cone (diameter 28 cm) was put in the center so that the airflow from the hairdryer (power 2000 W) fixed above the top of the cone was distributed uniformly. Air flow was set to its full and heating to the half of the maximal power. The translucent white lid covered the arena and diffused the light from a 75 W classical light bulb illuminating the scene from above in a 12 h light/12 h dark cycle. One centimeter gap was left between the lid and arena by three pegs so that the hot air stream may flow away (Supplementary Figure S2 right). The homogeneous light irradiance on the bottom of the arena was 0.22 W/m^2^ (measured by SKE500 and SKE510 system, Skye Instruments). The same light irradiance was set for the experiment with short wavelength part of the spectra (blue and green) filtered off. Long passing filter (FGL550S, Thorlabs) was used to let the yellow and red lights only (>550 nm) illuminate the scene. Spectra of lights (Supplementary Figure S3) were measured by the spectrometer USB 2000 (Ocean Optics). The camera (DTK21AU04, Imaging Source) was located 1 m under the transparent glass base with the dishes.

#### Training

**Figure 1:**
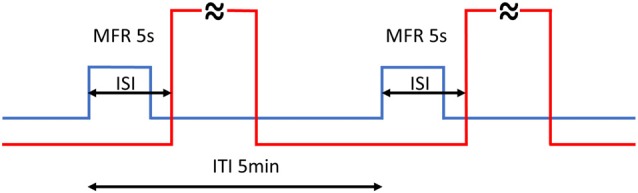
Timing of the training. Every rotation of magnetic North (MFR; blue line) lasted 5 s and was followed by 60 s hot air stream (red line—abridged). Back and forth rotations by 60° alternated periodically every 5 min (ITI). The whole training consisted of 20 training cycles in total, split into two blocks. The inter-stimulus intervals (ISIs) is the time between the start of MFR and onset of hot air stream. Different ISIs (−2, 0, 1, 2, 3, 5, 8 and 28 s) were applied.

Aversive training consisted of two blocks of GMF rotations by 60°: training A in the morning (8.30–9.15 am) and training B in the afternoon (2.30–3.15 pm), where every rotation was followed by a 60 s long stream of hot air (Figure 1). Under this regimen, every animal underwent 20 training cycles in total so that a learned link between GMF rotation and hot air stream could be made. Different ISIs (−2, 0, 1, 2, 3, 5, 8 and 28 s) and controls were subsequently applied in separate blocks (for the complete time list of experiments see Supplementary Table S1). The time switcher controlling CS and US synchronization could only be set in single second steps, hence no finer resolution than 1 s was technically feasible to attain among different ISIs.

#### Testing

Testing day followed each training day. Rotations of magnetic North by 60° back and forth alternating every 5 min were applied in an RF period between 11.30 and 1.00 pm. The rotations did not differ from the rotations used in the training day. As described previously (Vácha, 2006), the animal activity under the RF period was compared with their activity under the steady field baseline prior to and after the rotations.

### Data Evaluation

During testing day, images were captured every minute from 10.00 am to 2.30 pm. Altogether, 271 images from the testing day underwent statistical analysis. Custom-made automatic image analyzing software (RoachLab) evaluated individual animals’ movements. As a definition of movement, parameters were set as follows: body axis change by more than 15° or the shift of the body’s center by more than 2 cm between two subsequent images. The individual was discarded from the analysis if the SW detected the dish wall being touched more than 20 times (escaping behavior). Animals found dead the day after the test were also discarded from the evaluation. This way, 66 animals out of 1010 were excluded.

**Figure 2:**
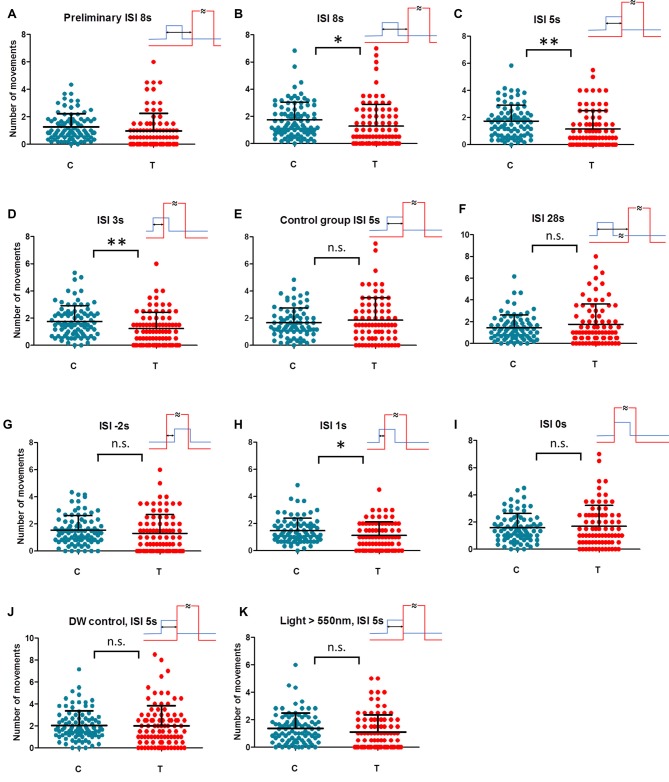
Individual movements under control (C) and magnetically treated (T) conditions in differently trained animals. Animal activity dropped (magnetically inducedfreezing, MIF) in trained animals with ISI = 8 s in the preliminary test **(A)**. The hypothesis of MIF was confirmed in the repeated sample **(B)**. No break between geomagnetic field (GMF) rotation (conditioned stimulus, CS) and hot air (unconditioned stimulus, US) in training (ISI = 5 s) gives the most prominent MIF in the test **(C)**. When CS and US partially overlap in ISI = 3 s and ISI = 1 s (**D,H** respectively) MIF is still significant. MIF disappears in simultaneous ISI = 0 s and also in reversed order training ISI = −2 s (**I,G** respectively). No MIF exists also in the case of too long a break between CS and US in ISI = 28 s **(F)**. In control samples, animals have been trained in ISI = 5 s but the coil was not fed at all on the next testing day **(E)** or double wrapped wiring hindered the GMF rotation **(J)** or short wavelength part of the light spectrum (<550 nm) was filtered off **(K)**. Each dot represents the number of movements of one animal per 30 min. Mean and SD (whiskers) are shown. Training time scheme is given in the upper-right for each condition (rotation of GMF—blue; hot air—red, ISI—two headed arrow). One asterisk: dependent *t*-test significance between rotating field (RF) and SF *P* < 0.05, two asterisks: *P* < 0.01. Detailed courses of each condition are given in complementary Supplementary Figure S4.

The whole dataset from the testing day was divided into nine time groups of 30 min each providing the time course of the behavior prior to and after the treatment (see Supplementary Figure S4). The analysis of data distribution within groups showed that the incidence of movement is rather intermittent (see primary data available online) and many zero values and generally non normal distribution prevent analysis of variance (ANOVA) application. Consequently, alternative, paired statistics had to be applied. The first three groups covered the data prior to magnetic rotations. The next three groups were magnetically treated (T) but this period could not simply be used as experimental as a whole. Since expected decline of learned reaction to GMF rotations without reinforcement by hot air really appeared (possibly due to the loss of motivation), only the first 60 min (groups 4 and 5) were chosen to reflect magnetically impacted behavior (see “Results” section and Supplementary Figure S4B). The group 6 which is still under RF but burdened by extinction of behavioral response had to be concerned separately (see “Results” section). Finally, the number of movements from groups 1–3 and 7–9 was pooled and used as a control (C) and compared with treated groups 4 and 5 (T). The sum of all movements for each respective animal was averaged per 30 min (Figure 2) for treated and control groups. Hence, individual pairs of movement numbers entered statistical evaluation. More abundant zero values in shorter (T) period would have biased nonparametric testing and hence, after checking the normal distribution of the individually taken differences between treated and control groups, a dependent (pair) *t*-test was applied (Statistica 10). In cases of different animal group evaluation a Mann-Whitney nonparametric test was used.

## Results

When naïve animals were exposed to a periodically rotated field, enhanced locomotor activity was apparent during the RF period (Supplementary Figure S4A)—a phenomenon described in our previous work (e.g., in Vácha, 2006; Bazalova et al., 2016) as magnetically induced restlessness (MIR). The onset of higher movement activity was delayed after the GMF rotation start and the restlessness persisted for several minutes after the magnetic treatment had ended (Supplementary Figure S4A). Such a phenomenon of delayed response is in line with other observations of magnetosensitive behavior (see “Discussion” section). Since this spontaneous reaction does not reflect transduction time, we employed an aversive conditioning technique in the next step.

We started with a preliminary test to see if a magnetically induced decline in activity (freezing) occurs in aversively trained animals at all. We also wondered how wide the time window after initiating magnetic treatment (T) may be considered as the critical period recognizable from control, baseline activity (C). Since the extinction of learned behavior or simply the loss of motivation are expected when CS is applied permanently without reinforcement (Engel and Hoy, 1999), we needed to set the time span narrow enough covering the conditioned reaction only. Figure 2A and Supplementary Figure S4B show the activity of animals exposed to GMF rotations trained aversively under ISI = 8 s a day before. In contrast to the naïve animals, where activity increases in the magnetically treated period (Supplementary Figure S4A and e.g., Vácha, 2006), the rapid decline of activity is apparent in trained animals and the term MIF may well be applied. Such a phenomenon is analogous to the conditioned immobility behavior responding to threats described in rodents and also in insects (Gibson et al., 2015; Takanashi et al., 2016). The MIF lasted about an hour even though the field still rotated every 5 min for the next half an hour in the period 6 (Supplementary Figure S4B). Based on this preliminary measurement, the individually-based comparisons between an average activity during 60 min of GMF rotations (T) vs. the average activity in the remaining time (C) was designated as a hypothetical parameter of magnetic sensitivity. However, the group 6 was still under RF but already not reflecting freezing behavior according to preliminary data (Supplementary Figure S4B). Due to this ambiguity it could not be considered either treated or control period and was excluded from statistical evaluation. The key hypothesis that movement in T (groups 4 and 5) differs from movement in C (groups 1–3 and 7–9) was ready to be tested.

In the following step, we repeated conditioning of trace type (time gap exists between CS and US) again at ISI = 8 s. Figure 2B and Supplementary Figure S4C showed a significant decline in activity (MIF) in the critical time (dependent *t*-test, *n* = 88, *P* = 0.0129). Hence, cockroaches could associate the magnetic cues with heat pulses and reduced the locomotion when magnetic rotation alone was applied. For all subsequent blocks of different ISIs and control experiments the same method was used.

Shortening the ISI to 5 s (Figure 2C and Supplementary Figure S4D) represented a conditioning of delay type (no break between CS and US). MIF was clear again (*n* = 82, *P* = 0.001) even more prominent than in the previous case.

Shortening the ISI to 3 s meant that the hot air stream started before the end of a 5 s long 60° GMF rotation. Nevertheless, MIF was still significant (Figure 2D and Supplementary Figure S4E; *n* = 83, *P* = 0.005).

As a following step, a control was scheduled when animals had been trained in ISI = 5 s but the GMF rotation was not applied on the next testing day (Figure 2E and Supplementary Figure S4F). MIF abatement (*n* = 75, *P* = 0.425) confirmed that the animals react to GMF specifically, and not to other factors such as the critical time period’s time of day.

Extending the ISI was scheduled as a subsequent experimental step. When animals were trained in ISI = 28 s, MIF was not present during the test. The course of activity (Figure 2F and Supplementary Figure S4G) shows insignificant elevation after beginning field rotation (*n* = 81, *P* = 0.203). Apparently, cockroaches could not keep the sensation of magnetic rotation in memory and associate it with aversive stimulus, now too delayed one after the other. Activity elevation suggests similarity with naïve animals’ reactions instead (Supplementary Figure S4A).

As a next step, we trained under ISI = −2 s where the order of CS and US was reversed and the onset of hot air preceded the start of magnetic field rotation by 2 s (Figure 2G and Supplementary Figure S4H). Under this setup, no MIF appeared (*n* = 84, *P* = 0.144) confirming the hypothesis that applying a strong and irritating US before CS distracts the attention of an animal from weak CS and prevents conditioning.

In the next step, we approached the crucial limit of ISI = 0 s. We concerned the risk that magnetoreception behavior may diminish in the course of testing from unknown reasons. This might give false results especially if control and treated groups are temporally separated as it was inherently in our case. To minimize the risk of this kind we alternated crucial ISI = 0 s and ISI = 1 s conditions within the period December 2016–January 2017 (see Supplementary Table S1) so that heterogeneous experimental scheme was ensured. The results show that if GMF rotation is advanced by only 1 s prior to hot air onset, it is sufficient for a conditioned response to appear (Figure 2H and Supplementary Figure S4I; *n* = 79, *P* = 0.011). However, under simultaneous onsets of GMF rotation and hot air in ISI = 0 s no association is created (Figure 2I and Supplementary Figure S4J; *n* = 78, *P* = 0.647). Hence, there is a time span between 0–1 s where the shortest functional ISI was found to be able to create a conditioned response to magnetic treatment.

At this point we incorporated the control measurement exploiting the double wrapped wiring of the coil. ISI = 5 s which had been found fully functional before was used, but no conditioned reaction appeared (Figure 2J and Supplementary Figure S4K; *n* = 93, *P* = 0.809). Consequently, the conceivable impacts of electric feeding itself, which might cause biasing sounds or coil vibrations (though it was extremely unlikely considering the coil construction and currents used) could be excluded.

Finally, to verify our hypothesis that MIF is really based on the mechanism dependent on the short wavelength light, hence likely linked to Cry-mediated magnetoreception as it was shown on magnetoreception of *P. americana* in our previous work (Bazalova et al., 2016), we adopted last control experimental measurement with optimal ISI = 5 s again but under long wavelength part of the spectrum only. After filtering off the blue and green part of the light spectrum (<550 nm) but keeping identical irradiance 0.22 W/m^2^, no significant MIF was found (Figure 2K and Supplementary Figure S4L; *n* = 90, *P* = 0.068).

Aside of pair testing presented up to this point, MIF was well apparent also when different animal groups tested in identical conditions were compared. While control periods don’t differ between ISI = 5 s and Control groups ISI = 5 s (Figure 2C vs. Figure 2E): Mann-Whitney *n* = 75.81; *P* = 0.735, the movement is limited in RF treated period compared to trained animals without RF (Figure 2C vs. Figure 2E): *n* = 75.81, *P* = 0.0036. Similarly, baseline activities don’t differ between ISI = 5 s and DW control (Figure 2C vs. Figure 2J): *n* = 91.81; *P* = 0.162, but the activity in treated period is higher if DW coil wiring prevents GMF from rotation (Figure 2C vs. Figure 2J): *n* = 91.81; *P* = 0.0013.

Although the experiments were performed during relatively long time period taking from April 2011 to March 2018 including 3 year break between 2013 and 2016 (see Supplementary Table S1), MIF response was apparent as stable phenomenon regardless of season.

## Discussion

It is the time delay between the sensory stimulus and neural response which provides important information about the way the energy of signal is transduced into membrane potential. According to Fogle et al. (2011), ectopic dmCry expression (*Drosophila* type of Cry) makes some fruit fly neurons light-responsive and stimulus—response delay is about 100 ms. Such relatively rapid membrane reaction shows that dmCry mediates a neural response to light *via* a pathway distinct from Cry’s known roles in circadian gene control (reviewed e.g., in Ozturk et al., 2011). Recently, Fogle et al. (2015) show that it is a redox sensor for the voltage-gated potassium channel that couples dmCry to rapid membrane depolarization and to acute behavioral response (Baik et al., 2017).

Cry-based reaction to light also turned out to be magnetosensitive. Ectopic dmCry expression conferred electrophysiologically detectable responsiveness to a magnetic field on *Drosophila* motoneurons (Giachello et al., 2016). With regard to the time scale, it took several tens of seconds before gradual membrane depolarization changes evoked by a constant blue light differed between magnetically treated and control conditions. However, the problem of how swift the magnetic stimulation is converted into a neural response has remained unanswered. According to our best knowledge, the speed of the Cry-based magnetoreception *in vivo* has never been recorded either electrophysiologically or otherwise.

A brief review of the transduction speed in cases of magnetoreception where no evidence of Cry involvement is available reveals the following: In-cell electrophysiology on a *Tritonia* sea slug showed an altered frequency of action potential in six neurons in the brain after periodic GMF rotation. The long latency (tens of minutes) after the application of rotation was notable (Lohmann et al., 1991; Popescu and Willows, 1999; Wang et al., 2004), indicating that they are locomotion-controlling efferent motor neurons (Cain et al., 2005) rather than primary receptor neurons. On the same species, the firing rates in single axons show substantial changes during the 10 min of stimulation, with a slow recovery over the next 20 min (Pavlova et al., 2011). Other studies report much shorter latencies between the magnetic stimulus and the onset of a neural response—within an interval of tens of milliseconds in trout (Walker et al., 1997) or birds (discussed in Vargas et al., 2006). However, Liedvogel and Mouritsen (2010) and Mouritsen and Hore (2012) warn that many older electrophysiological findings have turned out to be difficult to replicate due to likely biases accompanying the application of magnetic fields on tissue samples as well as artifactual stimulation of sensitive recording technique (Heyers et al., 2010).

There are recent reports of neural responses to magnetic treatment where authors hypothesize a Cry-independent reception mechanism based on iron oxide magnetic particles (mechanism reviewed in Shaw et al., 2015). Wu and Dickman (2012) show that the neural firing rate of the vestibular brainstem neurons in pigeons reflects a periodically rotating magnetic vector but the latency, and consequently the transduction time, haven’t been addressed. Selected examples of neural responses to the application of the field from the honeybee’s ventral nerve cord were shown by Liang et al. (2016), giving however little chance to distinguish between ON/OFF artifacts and a real sensory response. As a noninvasive indicator of magnetically induced neural activity in real time, Vidal-Gadea et al. (2015) used fluorescence detection of intracellular calcium in specific sensory neurons in nematode *Caenorhabditis elegans*. According to the records, the onset of neural response seems to be delayed much less than 1 s after the start of magnetic treatment.

Along with neural techniques, behavioral approaches seeking response latencies may provide important information towards understanding how magnetic information is transduced and processed in the brain (Vargas et al., 2006). Remarkably, delay lasting several minutes between the change in magnetic conditions and behavioral responses has been reported repeatedly: honeybees (Leucht and Martin, 1990), spiny lobsters (Lohmann et al., 1995), cockroaches (Vácha, 2006) and fruit flies (Bae et al., 2016). If caused by sensory transduction itself, such a long latency would hamper the steering of fast locomotion, especially over the short distances typical for many insect species. Several possible reasons for these latencies may stem from neural processing mechanisms (discussed e.g., in Wiltschko et al., 1998). While in naïve animals the behavioral reaction delay after a magnetic stimulus may take tens of minutes depending on their motivation and many other aspects, the timing of conditioning stimuli opens the way to understanding the rapidity of magnetic transduction. Therefore, our work adopted a conditioning approach to delve into this issue.

When exploring magnetic transduction delay, we utilized the fact that the time between the sensory cues (ISIs) defines if and how a conditioned link between them is made. The synchronization of two sensorial onsets—CS and US stimuli—makes sure that CS comes as a warning or notification shortly prior to US. ISI impacts the result considerably and event timing is crucial for successful associative learning (reviewed e.g., in Wasserman and Miller, 1997). In cases of classical forward conditioning, ISI is the time between CS and US onsets, which must not last too long so that the gap between cues still allows the animal to associate them. On the other hand, especially in aversive conditioning, ISI must not be equal to zero or negative because the overlapping incidence of electric shock or hot air stream along with a weaker sensory cue deafen its perception and the conditioned link does not emerge. The impact of US distracts the subject from perceiving the neutral CS (Wasserman and Miller, 1997). The best ISI for promoting a conditioned response is not a fixed point in time though. Optimal ISI = 3 s was found in the olfactory training of the sting extension reflex in honey bees (Giurfa et al., 2009) or ISI = 4 s in aversive visual conditioning of fruit-flies (Vogt et al., 2015). Tanimoto et al. (2004) report ISI = 23 s as the best for conditioned odor avoidance in *Drosophila*. However, when an aversive cue started simultaneously with olfactory stimulation or even before it, overlapping each other (so-called backward conditioning; negative ISI), no learning took place in honeybees (Giurfa and Malun, 2004; Giurfa et al., 2009). The same happened during aversive visual conditioning (Vogt et al., 2015) or aversive odor conditioning (Tanimoto et al., 2004; Murakami et al., 2010) in fruit-flies. Even in appetitive conditioning, no associative learning appeared in the *Spodoptera* moth if cues were presented simultaneously with ISI 0 s, and −3 s, −5 s or −10 s but was most prominent at ISI = 1 s (Fan et al., 1997). In line with previous cases, the CS needs to be presented 0–5 s before the onset of the US presentation to achieve appetitive olfactory learning in the cricket and disappears if ISI = −4 s is used (Matsumoto and Mizunami, 2002). Lent and Kwon (2004) investigated associative memory between visual and olfactory stimuli on *Periplaneta*, finding that if the light cue preceded the odor by ISI = 1 s, the conditioning was most effective. When US (electric shock) precedes CS (odor pulse), so called relief or backward conditioning may be successful again—but only if negative ISI is long enough and CS and US do not overlap (Tanimoto et al., 2004; Yarali et al., 2012; Vogt et al., 2015). Not only on organismal, but even on a synaptic facilitation level, it has been shown using the classical *Aplysia* model that ISI close to 0 s switches between functional and non-functional conditioning (Clark et al., 1994).

Considering our data on *Periplaneta*, when the gap between CS and US was too large (trace conditioning, ISI = 28 s, Figure 2F and Supplementary Figure S4G), cockroaches were not able to keep the information about magnetic rotation in mind and associate it with aversive stimulus delayed over the limit. Increasing the activity resembles naïve animal tests (Supplementary Figure S4A) instead. Because it was not our goal, we did not seek the upper ISI limit for successful trace conditioning; it falls between 8 s and 28 s in our experiments. It is well in line with visual conditioning in *Drosophila* (Tanimoto et al., 2004; Galili et al., 2011; Vogt et al., 2015), where the upper ISI limit in trace conditioning falls between 19 s and 34 s.

The manifestation of MIF in *Periplaneta* is the most significant at ISI lasting 5 s (Figure 2C and Supplementary Figure S4D), which is the only case when no time gap separates CS and US (delay conditioning) and CS and US do not overlap. Both shortening the ISI, which caused CS and US overlap (Figures 2D,H,I,G) and extending the time gap between them (Figures 2B,F) lead into decreased significance of conditioning test—in agreement with olfactory and visual trace conditioning (see above). To sum up the whole series of tests briefly: (i) MIF is behavior dependent on the short wavelength light (Figure 2K and Supplementary Figure S4L) as it was shown for spontaneous magnetoreception in our previous work (Bazalova et al., 2016), hence likely linked to Cry-mediated mechanism of reception; (ii) similarly to several other sensory pathway cases, there are distinct points in the ISI span where precise US and CS timing impacts pairing the magnetic and another event into memory record and causes a change in behavior.

Our work mainly seeks the speed of magnetoreception processes and is based on the idea that the transduction and transmission times of CS and US sensory inputs from sensory cells up to the brain must be close or equal if simultaneous application (ISI = 0 s) cancels learned pairing (discussed above). Besides, the definition of shortest functional ISI (long enough to be able to elicit a conditioned response) may help to learn how long it takes for two sensory stimuli to approach the brain. In order to elicit a conditioned reaction, US input (hot air) must reach the brain somewhat later than CS (magnet), at least by the shortest functional ISI. Such a condition can be expressed by a simple relation:

$$\begin{array}{l} {\text{T}}_{\text{US}}+{\text{ISI}}_{\text{SF}}-{\text{T}}_{\text{CS}}>0\text{ or}\\ {\text{T}}_{\text{US}}+{\text{ISI}}_{\text{SF}}>{\text{T}}_{\text{CS}} \end{array}$$

where T_US_ is the transduction and transmission time of hot air irritation; T_CS_ is the transduction and transmission time of the magnetic stimulus; and ISI_SF_ is the shortest functional ISI.

If the magnetic signal transfer (T_CS_) lasted longer than the summation T_US_ + ISI_SF,_ the magnetic information (CS) would reach the respective brain center *after* the time limit for successful pairing with the irritating hot air puff signal (US), which would *preclude making an association* between them.

Since neural processing of antennal afferents in *Periplaneta* is known and takes up to 100 ms (Mongeau et al., 2015), we assume that the hot air stroke is also transduced in the antennae and processed in the brain roughly within analogical T_US_ = 0.1 s. Besides, we have found MIF at ISI = 1 s and longer (ISI = 3, 5, 8 s) and no conditioned reaction at simultaneous ISI = 0 s and negative ISI = −2 s. Hence, ISI_SF_ must fall into the interval 0–1 s. Our conclusion concerning the arrival of CS and US information into the brain is: the delay caused by magnetic transduction in the receptor cell and transmission to the respective neural center takes place at a maximum of 1.1 s, likely much shorter.

Although *Periplaneta* is classical model organism for learning and memory studies (Mizunami et al., 2013) an intriguing question what part of an insect brain is responsible for pairing of magnetic and another stimuli is yet to be addressed.

Precisely measured transduction latency will possibly help to distinguish whether metabotropic or ionotropic transduction takes place in insect magnetoreception as was shown in the case of insect smell (Sato et al., 2008) or vision (Hardie and Raghu, 2001). Our behavioral assay doesn’t allow conclusions to be drawn with such precision, but it shows clearly that at least some cases of Cry-dependent magnetoreception are not based on regulation of gene expression or protein degradation, both of which are events known from Cry-regulated circadian rhythms (reviewed e.g., in Ozturk et al., 2011).

Our results are in line with the assumption of fast sensory transduction in magnetoreception typical for exteroreceptors in general. During high-speed locomotion, rapid transduction and processing of the information from sensory apparatus is a prerequisite for effective movement control feedback and takes from units to hundreds of milliseconds (Szyszka et al., 2014; Mongeau et al., 2015; Bhavsar et al., 2017). Cry’s recently reported role in arousal responses and *Drosophila* phototaxis (Baik et al., 2017) may point to a common Cry-mediated swift signaling mechanism.

We admit that results of our assay don’t necessarily reflect the way the magnetic information is used by free-living cockroaches for spatial orientation. In natural conditions, summation or averaging of minute magnetic changes when position of magnetic axis is sought might take longer. Nevertheless, the results of this assay show that magnetic signal transduction, its transmission into the central nervous system and the creation of a conditioned link take place within a time range comparable to other senses—an aspect of magnetoreception generally expected but not verified to date. Hence, the magneto-sensitive signaling pathway based on Cryptochrome is sufficiently fast to control rapid insect locomotion and may steer its orientation in space.

## Data Accessibility

Data reported in this manuscript are deposited at: https://is.muni.cz/www/1376/71063192/ISI_Primary_Data_Vacha.xlsx.

## Ethics Statement

The authors declare that the present study was carried out in accordance with the current laws of the Czech Republic as well as ACPA and does not require ethics approval.

## Author Contributions

PS, RN, JK and MV conceived the study and wrote the first draft. KT, PB, PS, RN and JK collected data. PS, JK and RN analyzed data. MV wrote the final article.

## Conflict of Interest Statement

The authors declare that the research was conducted in the absence of any commercial or financial relationships that could be construed as a potential conflict of interest.
