# Supplementary material for: How Swift Is Cry-Mediated Magnetoreception? Conditioning in an American Cockroach Shows Sub-second Response
Source: Front Behav Neurosci. 2018 May 28;12:107. doi: 10.3389/fnbeh.2018.00107 (PMC5985609; doi:10.3389/fnbeh.2018.00107)
Supplement: Supplementary file 1 [file Data_Sheet_1.pdf]

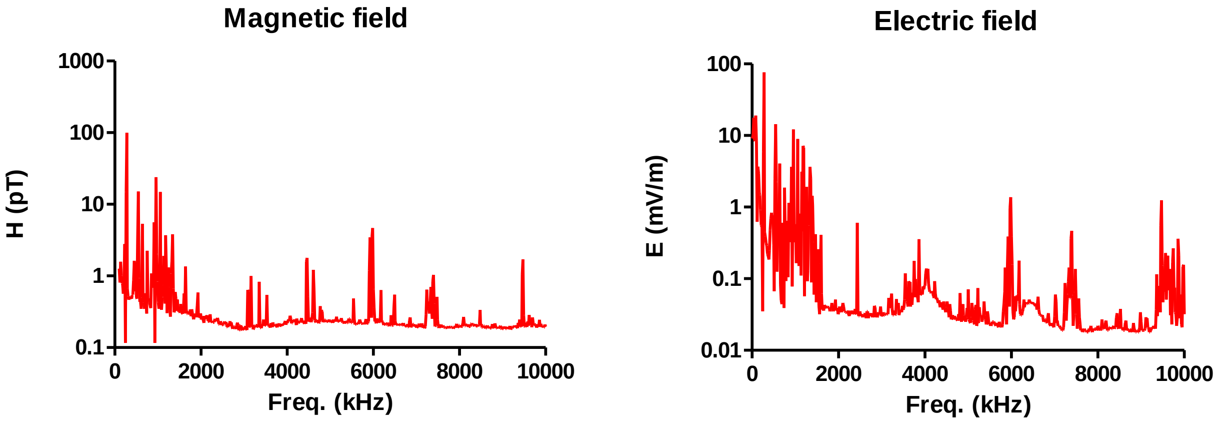


**Supplementary Fig. S1**: **Radiofrequency background in the laboratory.** Magnetic component is given on the left and electric on right. Measurement was set to make it comparable with Schwarze et al., (2016). A range between 9 kHz and 10 MHz was measured. Calibrated 6511 Lindgren passive antenna for magnetic component and active antenna Schwarzbeck EFS 9218 for electric component were used. Spectrum analyser R&S FSC3 was set for 30 min of max-hold intensity measurement with a resolution bandwidth of 10 kHz.
Magnetic intensity (flux density - H) summed over all the frequencies in the spectra (calculated according to Schwarze et al., 2016) was 0.6 nT.

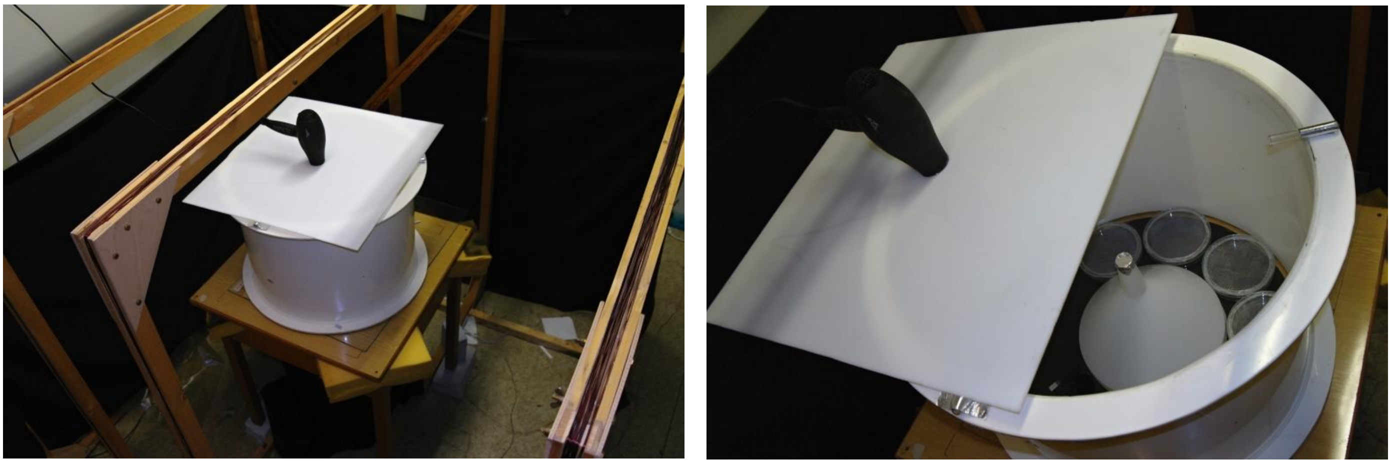

**Supplementary Fig. S2: Testing arrangement.** Left picture: the arena was placed on the wooden table in the centre of the Merritt coil. The translucent white lid covered the arena and hairdryer was fixed in the centre. Right picture: Petri dishes were covered by lids with grids made of wire. Plastic cone in the centre distributed the airflow from the hairdryer uniformly. The camera (not shown) was located on the floor 1 m under the transparent glass base with the dishes. Wooden stud strengthening the coils system as well as black cloth covering the coil were removed due to taking the pictures.

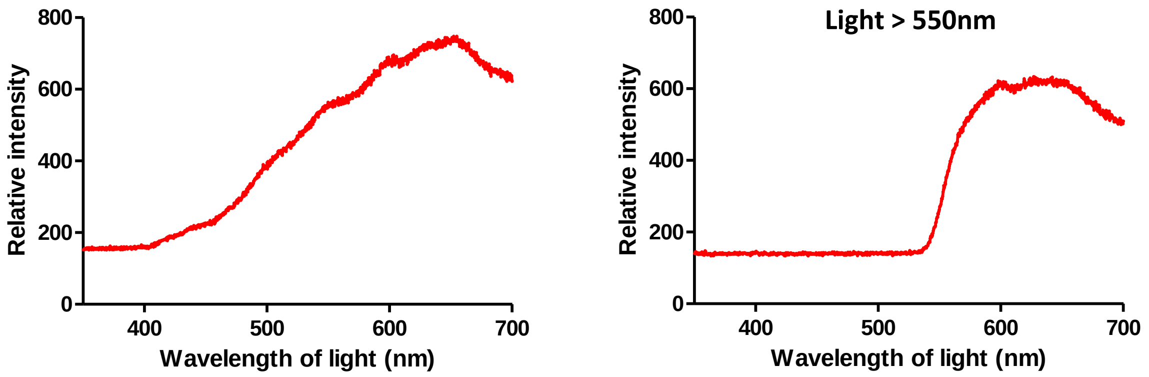


**Supplementary Fig. S3**: **Light spectra in the laboratory.** Full spectrum from the light bulb is given on the left and after filtering the short wavelength part of the spectra off on the right. Filtered light spectrum was used in the experiment “Light > 550nm, ISI 5s”. The irradiance was set to be equally 0.22 $W/m^2$ in both cases.

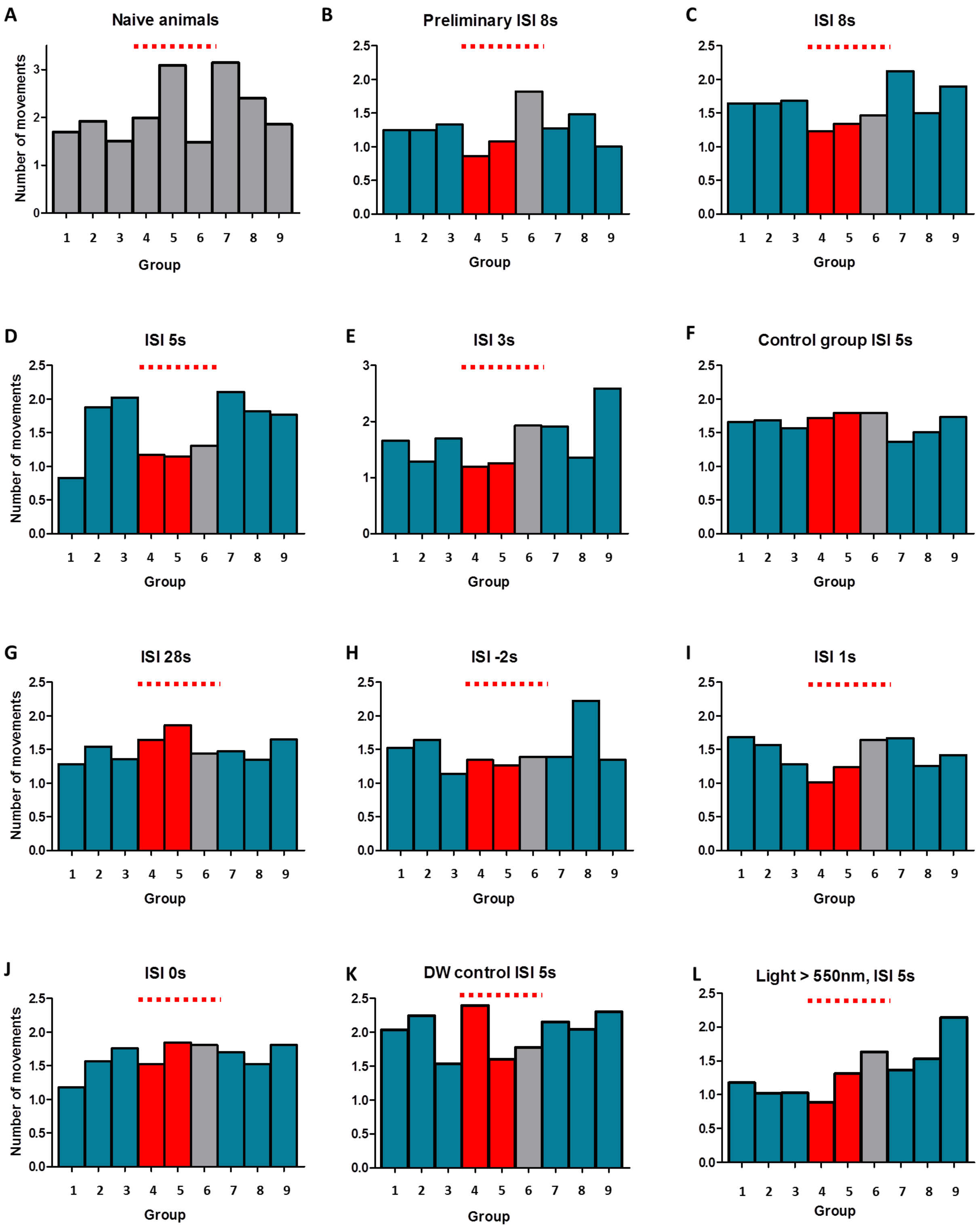


**Supplementary Fig. S4: Pooled movements of all animals in the course of testing, being trained under different ISIs.** Testing started a day after the training at 10.00 am and finished at 2.30 pm. The whole dataset was divided into 9 groups of 30 min each. Groups 4-6 underwent magnetic treatment (periodical rotations from 11.30 am to 1.00 pm). Reaction of naïve animals (A) is shown only to illustrate the typical behaviour compatible with our previous results (Vácha, 2006) where elevation of activity follows the onset and cessation of the magnetic rotations. However, drop of the activity is apparent when aversively trained animals are used (B). Since the extinction of learned magnetically stimulated freezing without reinforcement was definable in the preliminary test (B), only the first 60

minutes (groups 4 and 5) were chosen for further MIF statistical evaluation. Since group 6 could not be considered either treated or control period exclusively it was excluded from statistical evaluation (grey bar), groups 1-3 and 7-9 were pooled and used as controls (blue bars) and compared with treated pooled groups 4 and 5 (red bars). For comments on each condition (C-L) see the legend to complementary Fig 2. Mean values of activity are given; GMF rotations illustrated as red broken line.

**Supplemetary Table 1:** Chronologically ordered list of experiments. Experiments with significant MIF are given in red.

| Date | Conditions | Date | Conditions | Date | Conditions | Date | Conditions |
|---|---|---|---|---|---|---|---|
| 22.4.2011 | Naive animals | 20.3.2013 | ISI 5s | 18.9.2013 | ISI 28s | 31.5.2017 | DW control ISI 5s |
| 4.5.2011 | Naive animals | 22.3.2013 | ISI 5s | 9.10.2013 | ISI 28s | 2.6.2017 | DW control ISI 5s |
| 5.11.2011 | Naive animals | 29.3.2013 | ISI 5s | 11.10.2013 | ISI 28s | 4.6.2017 | DW control ISI 5s |
| 11.11.2011 | Naive animals | 5.4.2013 | ISI 5s | 13.10.2013 | ISI 28s | 7.6.2017 | DW control ISI 5s |
| 12.11.2011 | Naive animals | 7.4.2013 | ISI 5s | 18.10.2013 | ISI 28s | 9.6.2017 | DW control ISI 5s |
| 25.11.2011 | Naive animals | 17.4.2013 | ISI 5s | 11.12.2013 | ISI 28s | 11.6.2017 | DW control ISI 5s |
| 4.1.2012 | Naive animals | 19.4.2013 | ISI 5s | 13.12.2013 | ISI 28s | 14.6.2017 | DW control ISI 5s |
| 30.11.2012 | ISI 28s | 24.4.2013 | ISI 5s | 24.10.2016 | ISI -2s | 26.9.2017 | DW control ISI 5s |
| 2.12.2012 | preliminary ISI 8s | 3.5.2013 | ISI 5s | 26.10.2016 | ISI -2s | 28.9.2017 | DW control ISI 5s |
| 5.12.2012 | preliminary ISI 8s | 5.5.2013 | ISI 5s | 28.10.2016 | ISI -2s | 6.10.2017 | DW control ISI 5s |
| 12.12.2012 | preliminary ISI 8s | 24.5.2013 | ISI 3s | 2.11.2016 | ISI -2s | 8.10.2017 | DW control ISI 5s |
| 14.12.2012 | ISI 28s | 26.5.2013 | ISI 3s | 4.11.2016 | ISI -2s | 28.2.2018 | >550nm ISI 5s |
| 17.12.2012 | ISI 28s | 31.5.2013 | ISI 3s | 6.11.2016 | ISI -2s | 2.3.2018 | >550nm ISI 5s |
| 21.12.2012 | preliminary ISI 8s | 2.6.2013 | ISI 3s | 10.11.2016 | ISI -2s | 4.3.2018 | >550nm ISI 5s |
| 6.1.2013 | preliminary ISI 8s | 5.6.2013 | ISI 3s | 12.11.2016 | ISI -2s | 6.3.2018 | >550nm ISI 5s |
| 9.1.2013 | preliminary ISI 8s | 7.6.2013 | ISI 3s | 17.11.2016 | ISI -2s | 8.3.2018 | >550nm ISI 5s |
| 11.1.2013 | preliminary ISI 8s | 9.6.2013 | ISI 3s | 19.11.2016 | ISI -2s | 14.3.2018 | >550nm ISI 5s |
| 13.1.2013 | preliminary ISI 8s | 14.6.2013 | ISI 3s | 11.12.2016 | ISI 0s | 16.3.2018 | >550nm ISI 5s |
| 16.1.2013 | preliminary ISI 8s | 17.6.2013 | ISI 3s | 13.12.2016 | ISI 0s | 18.3.2018 | >550nm ISI 5s |
| 3.2.2013 | preliminary ISI 8s | 19.6.2013 | ISI 3s | 15.12.2016 | ISI 1s | 20.3.2018 | >550nm ISI 5s |
| 8.2.2013 | ISI 8s | 10.7.2013 | Control group ISI 5s | 17.12.2016 | ISI 1s | 23.3.2018 | >550nm ISI 5s |
| 13.2.2013 | ISI 8s | 12.7.2013 | Control group ISI 5s | 19.12.2016 | ISI 1s | 25.3.2018 | >550nm ISI 5s |
| 16.2.2013 | ISI 8s | 17.7.2013 | Control group ISI 5s | 23.12.2016 | ISI 0s | | |
| 20.2.2013 | ISI 8s | 20.7.2013 | Control group ISI 5s | 25.12.2016 | ISI 0s | | |
| 22.2.2013 | ISI 8s | 24.7.2013 | Control group ISI 5s | 29.12.2016 | ISI 0s | | |
| 24.2.2013 | ISI 8s | 26.7.2013 | Control group ISI 5s | 4.1.2017 | ISI 0s | | |
| 27.2.2013 | ISI 8s | 28.7.2013 | Control group ISI 5s | 6.1.2017 | ISI 0s | | |
| 3.3.2013 | ISI 8s | 31.7.2013 | Control group ISI 5s | 8.1.2017 | ISI 1s | | Significant MIF |
| 8.3.2013 | ISI 8s | 12.8.2013 | Control group ISI 5s | 10.1.2017 | ISI 1s | | Controls or no MIF |
| 13.3.2013 | ISI 8s | 17.8.2013 | Control group ISI 5s | 12.1.2017 | ISI 1s | | |
| | | 24.8.2013 | Control group ISI 5s | 14.1.2017 | ISI 1s | | |
| | | | | 16.1.2017 | ISI 1s | | |
| | | | | 18.1.2017 | ISI 1s | | |
| | | | | 25.1.2017 | ISI 0s | | |
| | | | | 27.1.2017 | ISI 0s | | |

**Supplementary References:**


1. Schwarze S, Schneider N-L, Reichl T, Dreyer D, Lefeldt N, Engels S, Baker N, Hore PJ, Mouritsen H. 2016. Weak Broadband Electromagnetic Fields are More Disruptive to Magnetic Compass Orientation in a Night-Migratory Songbird (Erithacus rubecula) than Strong Narrow-Band Fields. Frontiers in Behavioral Neuroscience 10(55):doi: 10.3389/fnbeh.2016.00055.
2. Vácha M. 2006. Laboratory behavioural assay of insect magnetoreception: magnetosensitivity of Periplaneta americana. J Exp Biol 209:3882-3886.
